# Effects of refractive accommodation on subfoveal choroidal thickness in silicone oil-filled eyes

**DOI:** 10.1186/s12886-022-02332-y

**Published:** 2022-03-05

**Authors:** Ying Yan, Ran Liu, Chengyuan Gao, Yanping Song, Qin Ding, Feng Chang, Xiao Chen

**Affiliations:** 1 Department of Ophthalmology, Middle Theater General Hospital, 627 Wuluo Road, Wuhan, 430070 China; 2 Southern Medical University, Guangzhou, China

**Keywords:** Choroidal vascularity index, Optical coherence tomography, Silicone oil, Subfoveal choroidal thickness

## Abstract

**Purpose:**

To investigate the effects of refractive accommodation on subfoveal choroidal thickness (SFCT) in silicone oil (SO)-filled eyes.

**Methods:**

This retrospective, self-comparative study was conducted on 40 patients with unilateral macula-on rhegmatogenous retinal detachment, who underwent vitrectomy and SO tamponade. The SFCT of SO-filled eyes and the fellow control eyes were measured using optical coherence tomography at their one-month visit after surgery. The patients wore soft contact positive lenses for 24 h in the SO-filled eyes, to correct their refractive error. SFCT and choroidal vascularity index (CVI) were measured before and after wearing the contact lenses. Mean SFCT was compared between SO-filled eyes and the fellow control eyes, and SFCT and CVI were compared before and after refractive error correction in the SO-filled eyes.

**Results:**

Mean SFCT of SO-filled eyes (221.52 ± 38.41 um) was less than that of the fellow eyes (273.41 ± 31.30 um) (*P* < 0.001). After refractive error correction, the mean SFCT increased to 269.28 ± 36.90 um(*P* < 0.001). However, CVI decreased from 57.01 ± 2.41 to 55.39 ± 2.39 (*P* < 0.05).

**Conclusions:**

SFCT reduction in SO-filled eyes was primarily due to the hyperopia status. The non-uniform change in CVI suggests that changes in CT are mainly attributed to a greater expansion of the stromal area instead of the choroidal vascular area.

**Trial registration:**

This study protocol was reviewed and approved by the Ethics Committee of the Central Theater General Hospital, approval number No. [2020]058–1, retrospectively registered.

## Background

Silicone oil (SO) has been used as an intraocular tamponade, since many years in vitreoretinal surgery, and its toxic effects on retina have been reported through several studies [1–4]. Some recent studies have reported that SO leads to a reduction in choroidal thickness (CT) [5, 6], which can also be considered a toxic effect on choroidal tissues [5, 7]. However, SO induced refractive error (RE) (usually present in hyperopia), which can considerably influence the CT value, has not been adequately evaluated in these studies. As the refractive state could have a substantial impact on CT [8–10], we hypothesized that the RE induced by SO tamponade has an impact on the CT.

In the present study, we measured the SFCT of the SO-filled eyes and fellow control eyes and compared the changes in SFCT after the correction of RE in the SO-filled eyes. In addition, we analyzed choroidal vascularity index (CVI) that seems to be a robust marker [11] to assess choroidal vascular status in these patients.

## Methods

### Patients

This retrospective, interventional comparative study was conducted on 40 patients with unilateral macula-on rhegmatogenous retinal detachment, recruited from the Department of Ophthalmology at Central Theater General Hospital. The fellow 40 eyes were served as control. The patients were successfully treated with a three-port pars plana vitrectomy (PPV) without internal limiting membrane (ILM) peeling and a 5,700-cSt SO endotamponade (Bausch&Lomb, New York,United States of America).

Exclusion:

- Patients with documented previous ocular disease (glaucoma, diabetic retinopathy, macular degeneration
- high myopia exceeding -6.0 diopters
- with other than uncomplicated cataract surgery
- Macular-off RRD
- Received more than one surgery repaired
- With PVR grade 3 or 4 and eceived retinotomy
- with choroidal detachment
- Intraocular pressure greater than 21 mmHg
- Considering intraocular pressure [12] and antiglaucomatous drops [13] had a significant effect on SFCT, we excluded the patients postoperative intraocular pressure more than 21 mmHg.

This study was approved by the institutional review board at Central Theater General Hospital and was conducted in accordance with the Declaration of Helsinki. All the patients were informed of the nature of their disease and of all potential treatment options, and informed consents were obtained.

A complete ocular examination was conducted in each patient, including the determination of best-corrected visual acuity (BCVA) using standard Snellen eye charts, RE screened with autorefractor (Canon Autorefractor RK-F1, Canon Inc. Ltd., Tochigiken, Japan), measurement of intraocular pressure (IOP), slit-lamp examination, stereoscopic observation of the fundus, and macular measurements using swept-source optical coherence tomography (SS-OCT) (DRI OCT-1 Atlantis, TOPCON Corp, Tokyo, Japan).

### Optical Coherence Tomography Measurements

SFCT was measured using SS-OCT. According to the guidelines suggested by Kikushima [14], the quality of OCT images was automatically evaluated by Topcon image quality factor, and scored from 0 to 100. Only high-quality images (quality factor ≥ 60) were used for analysis.

**Fig. 1:**
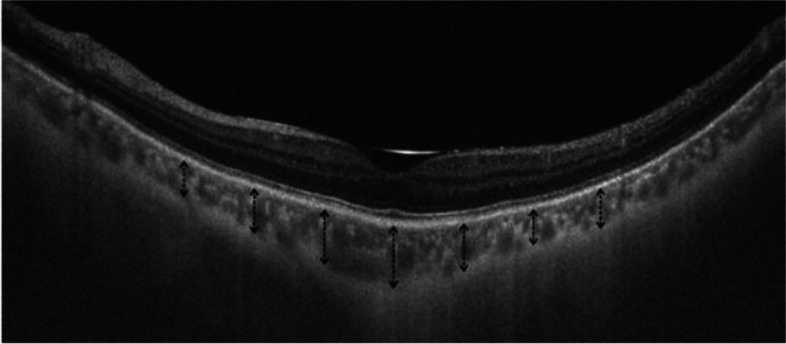
A representative SS-OCT measurement of SFCT. Black dashed arrows indicate an interval of 0.5 mm from the fovea up to 1.5 mm choroidal thickness

CT was defined as the distance between the base of the subfoveal retinal pigment epithelium and the margin of the choroidoscleral interface. SFCT was measured at the fovea, and at 500-um distances up to 1.5 mm, temporal and nasal to the fovea in horizontal direction, and up to 1.5 mm, superior and inferior to the fovea in vertical direction using the manual segmentation method (shown in Fig. 1). The mean overall SFCT was obtained by calculating the average value of SFCT measurements at all eccentricities.

**Fig. 2:**
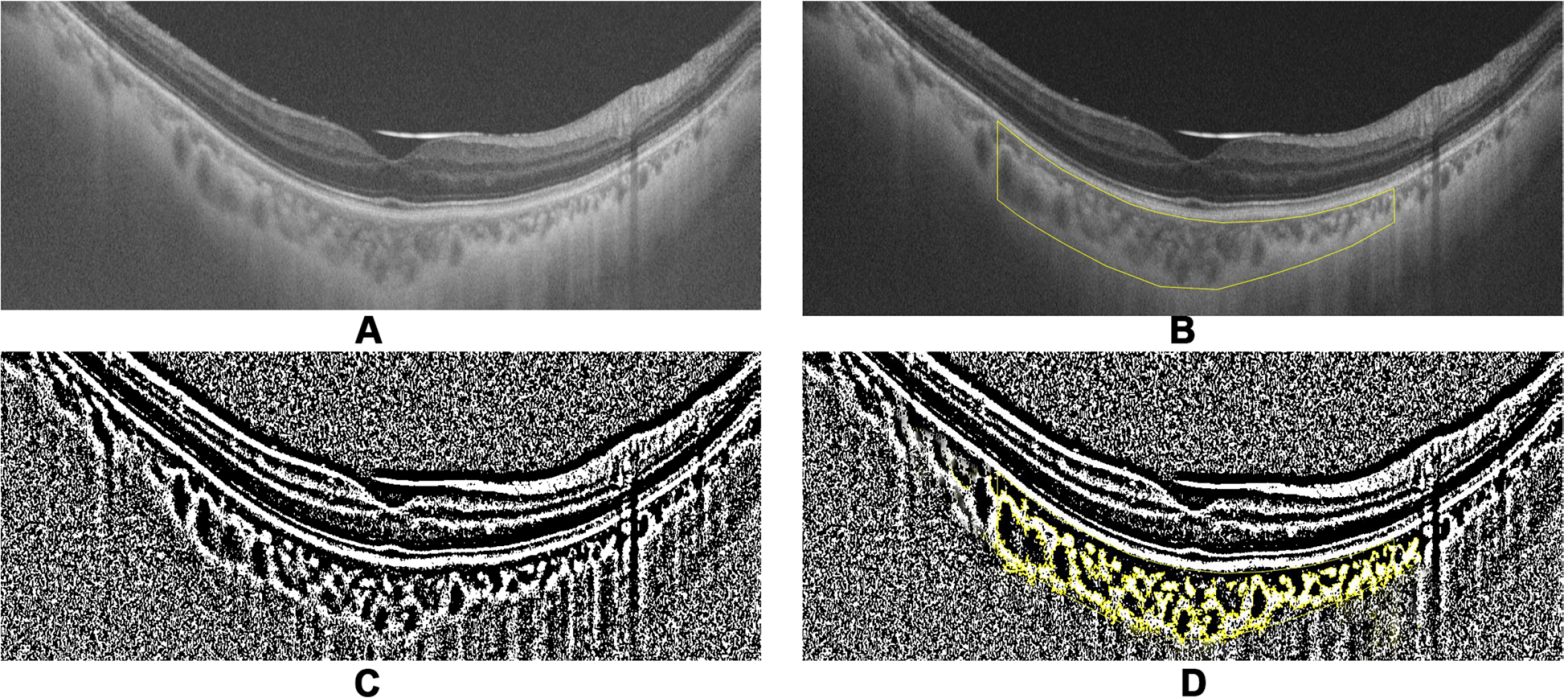
A Representative image processing to obtain CVI and choroidal vascular area. **A** Original SS-OCT image. **B** A 1.5-mm segmentation block of the subfoveal choroidal area using the polygon selection tool. **C** Segmented OCT image using a modifified image binarization approach. **D** The vascular area was highlighted by applying the color threshold. The ratio of the vascular area to the total choroidal area was termed the CVI

CVI, defined as the proportion of vascular area to the total choroidal area, was measured on the SS-OCT images [15, 16]. In brief, after uploading the images on the ImageJ software (http://imagej.nih.gov/ij/; provided in the public domain by the National Institute of Health), they were converted to 8-bit images to allow application of the autothreshold. A 3000-um thick line centered at the fovea was drawn. The part of choroid beneath the line was segmented, and total subfoveal choroidal area was computed. The auto local threshold was determined using the Niblack auto local threshold to demarcate the choroidal vascular area and stromal area (shown in Fig. 2).

The OCT scans were performed in both eyes to measure SFCT and CVI, one month after surgery. The SO-filled eyes wore soft, positive, contact lenses (Kushi, China) for 24 h to correct the RE, depending on its optometry value. SFCT and CVI were measured once again, before removing the contact lenses. Mean SFCT and CVI were compared before and after wearing lenses in SO-filled eyes. Previous studies have shown that the repeatability of the CT and CVI measurements were excellent [17, 18]. In order to enhance patient compliance, only a single examination was performed by SS-OCT each time. Considering the diurnal variation in CT, all examinations were performed between 9 a.m. and 10 a.m.The measurements were performed manually by two retina specialists using calipers provided with the device, and the averaged values were saved and considered for statistical analysis.

### Statistical analyses

All statistical analyses were performed with a commercial analytical package (SPSS Statistics 22.0 for Windows; SPSS, Inc., IBM, Somers, NY, USA). Continuous variables were expressed as mean ± standard deviation. Paired t -tests and unpaired t-tests were used to analyze numerical and ordinal variables, respectively. A *P* value < 0.05 was considered to be statistically significant.

## Results

Twenty-five men and fifteen women (mean age, 38.47 ± 8.02 years; range, 20–50 years) were enrolled. Anatomical success (complete retinal reattachment) was noted in all cases. Mean RE of the SO-filled eyes was + 6.64 ± 1.25 D, ranging from + 4.5 to + 9.5 D. Mean preoperative BCVA was 20/125, ranging from 20/200–20/50 (logarithm of the minimum angle of resolution (logMAR) was 0.81 ± 0.28).

The mean BCVA 1 month after surgery was 20/63, ranging from 20/100–20/25 (logMAR was 0.49 ± 0.22) (*P* < 0.001). The mean preoperative IOP was 12.3 ± 4.2 mmHg, and mean IOP 1 month after surgery was 16.4 ± 3.5 mmHg (*P* > 0.05). Demographic and clinical characteristics of patients are summarized in Table 1.

**Table 1:** Demographic and Clinical Characteristics of Patients

Variables	Mean	SD	Range
Patients’age (years)	38.47	8.02	20–50
Duration of symptoms(weeks)	3.29	2.20	1–8
Preoperative BCVA			
logMAR (a)	0.32	0.14	0.1–0.6
Snellen, median	20/40		20/80–20/25
BCVA 1 months after surgery			
logMAR (b)	0.12	0.07	0.0–0.2
Snellen, median	20/25		20/32–20/20
Preoperative IOP (mmHg) (c)	12.3	4.2	8.0–16.2
IOP 1 months after surgery (mmHg) (d)	16.4	3.5	13.1–21.0
RE of the eyes after SO tamponaded (diopters), mean ± SD	+ 6.64	1.25	+ 4.5- + 9.5

*P* < 0.001, for (a) Versus (b) Comparison.*P* > 0.05, for (c) Versus (d) Comparison.The Wilcoxon matched-pairs signed rank test was performed

**Fig. 3:**
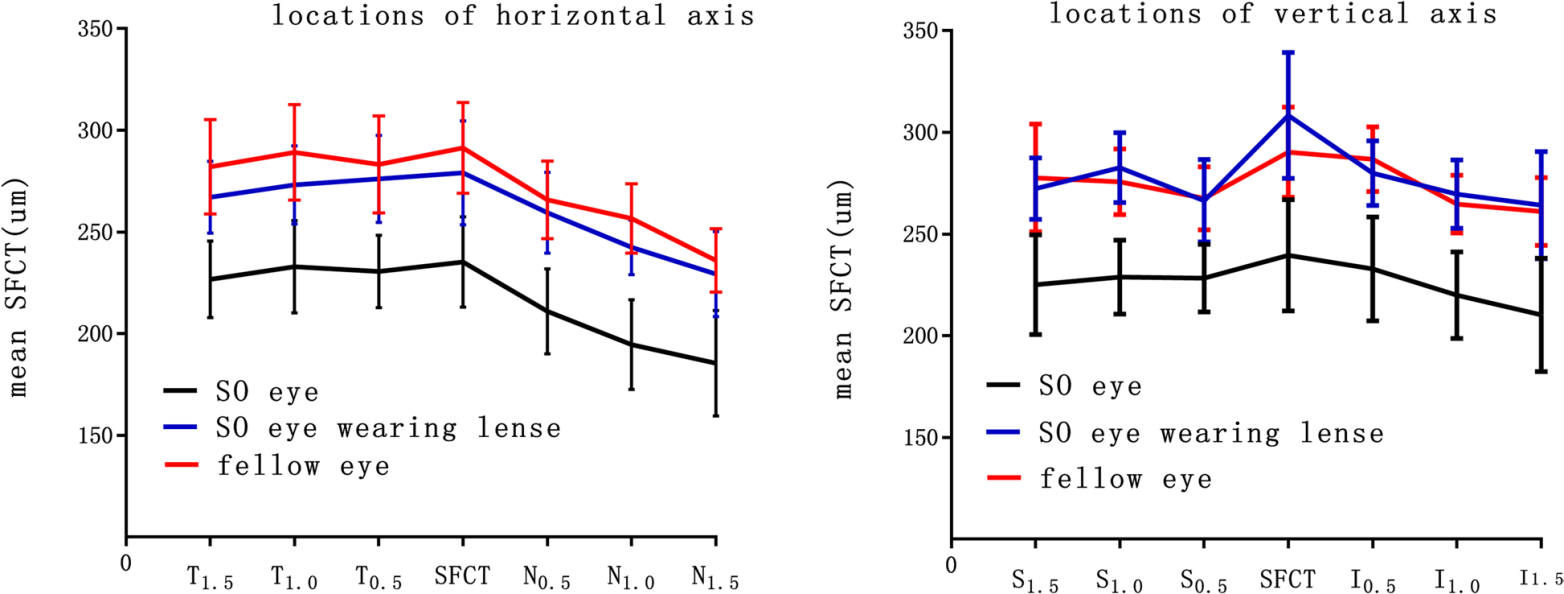
Graph showing the differences in three groups (followed eyes, SO tamponaded eyes before and after lenses wore) along the horizontal axis and vertical axis

It was observed that the mean SFCT of SO-filled eyes was 221.52 ± 38.41 um, mean SFCT of the fellow eyes was 273.41 ± 31.30 um, and SFCT of SO-filled eyes was significantly less than that of the fellow eyes, 1 month after SO tamponade (*P* < 0.001). Mean SFCT of SO-tamponaded eyes after wearing lenses was 269.28 ± 36.90 um. Mean SFCT of SO-tamponaded eyes significantly increased by wearing positive contact lenses (*P* < 0.001) (shown in Fig. 3).

However, CVI decreased from 57.01 ± 2.41 to 55.39 ± 2.39 (*P* = 0.044) after RE correction.

## Discussion

In this study, we observed that SFCT of SO-filled eyes was significantly less than that of the fellow control eyes, 1 month after vitrectomy and SO tamponade. Similar results were obtained by Odroniba et al. and Karimi et al. [5], who reported that SFCT decreased after PPV and SO tamponade. Moreover, Saeed Karimi et al. [19], reported that 3 months after SO removal, the mean SFCT Increased only about 2 µm, which is not clinically significant, leading to the conclusion that SO-induced choroid thinning did not improve after SO removal. Sugawara et al. [6] showed that CT of eyes with retinal detachment was similar to the fellow eyes before treatment. Therefore, previous studies have shown that PPV, by itself, did not have any significant effect on CT [20]. These findings suggest that the reduction in SFCT of SO-filled eyes after PPV might be related to the negative effects of intravitreal SO.

However, the possible mechanism of SO tamponade leading to a decrease in choroid thickness is not clearly understood. Some authors have proposed that SO toxicity might be caused by a failure of potassium siphoning by the Müller cells [21]. Another mechanism by Odrobina D suggested that SO could induce a type of microangiopathy in the retina and choroid, leading to altered blood flow and ischemia, subsequently causing retinal and choroidal thinning [5]. However, the mechanism has not been proved scientifically.

As early as in 1995, an interesting study performed on chickens by Wallman et al. [8, 9] showed that spectacle lens-induced hyperopia and myopia mediated the changes in thickness of the choroid. The choroid can increase its thickness in response to myopic defocus (image focused in front of the retina) by pushing the retina towards the image plane. On the contrary, choroidal thinning is observed in response to hyperopic defocus (image focused behind the retina) [22]. A similar choroidal mechanism for regulation of the refractive state has been proved to exist in humans as well [10].

Based on these observations and the results of our study, we believe that the reduction in SFCT of SO-filled eyes moved the retina backward, bringing the photoreceptors into plane of focus. In contrast, increased SFCT can occur due to the backward movement of the focal plane by wearing positive lenses.To the best of our knowledge, this is the first study to investigated the effects of refractive accommodation on SFCT in SO-filled eyes.Results indicated SFCT reduction in SO-filled eyes was primarily due to the hyperopia status.

The choroid is a vascular layer that supplies oxygen and nutrients to the outer part of retina [23]. Choroidal thinning may lead to a relative decrease in choroidal circulation, eventually leading to a reduced level of necessary oxygen and nutrient delivery to the most metabolically active foveal region [24].

In contrast to CT, CVI, defined as the proportion of the luminal area to the total choroid area [11],shows lesser variability, being influenced by fewer physiological factors such as axial length, RE, intraocular pressure, systolic blood pressure, and diurnal variation [25]. It can help in understanding the variations in vascularity of chorioretinal compartments through simultaneously obtained images by OCT [26].

In our study, CVI of SO-filled eyes decreased after RE. After thoroughly analyzing our results, we concluded that the increase in thickness could be attributed to an expansion of the stromal area instead of choroidal vascular area. The theory is supported by a research conducted by Wallman, which stated that the oblique or tangential arrangement of the non-vascular smooth muscles between the lacunae makes it plausible that they might play a role in the changes in CT occurring in response to retinal defocus [27].

The principal limitations of this study include the relatively small sample size. We did not perform the SS-OCT measurement before PPV and after SO removal, and the SFCT was measured just 1 month after PPV. Nonetheless, the results can help in understanding the influence of hyperopia caused by SO on CT. In future, long-term prospective studies, involving a larger population, and a longer follow-up before and after SO removal would be needed to improve our clinical understanding of CT and vascularity of choroid.

In conclusion, the reduction in SFCT of SO-filled eyes may be primarily due to the hyperopia caused by SO, which can be reversed after RE correction. The non-uniform change in CVI suggests that changes in CT are mainly attributed to an expansion of the stromal area instead of choroidal vascular area.

## Data Availability

The datasets used and/or analyzed during the current study are not publicly available due to their containing information that could compromise the privacy of research participants, but are available from the corresponding author Xiao Chen(cxfn817@163.com) on reasonable request. This work was completed at the Department of Ophthalmology in Central Theater General Hospital, Wuhan, China. Consent to Publish.

## Abbreviations

BCVA: Best-corrected visual acuity
CT: Choroidal thickness
CVI: Choroidal vascularity index
IOP: Intraocular pressure
PPV: Pars plana vitrectomy
RE: Refractive error
SFCT: Subfoveal choroidal thickness
SO: Silicone oil
SS-OCT: Swept-source optical coherence tomography
